# IgM antibody responses against *Plasmodium* antigens in neotropical primates in the Brazilian Atlantic Forest

**DOI:** 10.3389/fcimb.2023.1169552

**Published:** 2023-09-27

**Authors:** Gabriela Maíra Pereira de Assis, Denise Anete Madureira de Alvarenga, Luisa Braga e Souza, Juan Camilo Sánchez-Arcila, Eduardo Fernandes e Silva, Anielle de Pina-Costa, Gustavo Henrique Pereira Gonçalves, Júlio César de Junior Souza, Ana Julia Dutra Nunes, Alcides Pissinatti, Silvia Bahadian Moreira, Leticia de Menezes Torres, Helena Lott Costa, Herlandes da Penha Tinoco, Valéria do Socorro Pereira, Irene da Silva Soares, Taís Nóbrega de Sousa, Francis Babila Ntumngia, John H. Adams, Flora Satiko Kano, Zelinda Maria Braga Hirano, Lilian Rose Pratt-Riccio, Cláudio Tadeu Daniel-Ribeiro, Joseli Oliveira Ferreira, Luzia Helena Carvalho, Cristiana Ferreira Alves de Brito

**Affiliations:** ^1^ Grupo de Pesquisa em Biologia Molecular e Imunologia da malária, Instituto René Rachou/Fiocruz Minas, Belo Horizonte, Brazil; ^2^ School of Natural Sciences, Molecular and Cell Biology Department, University of California, Merced, Merced, CA, United States; ^3^ Vice-diretoria de Ensino, Instituto René Rachou/Fiocruz Minas, Belo Horizonte, Brazil; ^4^ Laboratório de Doenças Febris Agudas, Instituto Nacional de Infectologia Evandro Chagas (INI), Fiocruz, Rio de Janeiro, Brazil; ^5^ Centro de Pesquisa, Diagnóstico e Treinamento em Malária (CPD-Mal), Instituto Oswaldo Cruz, Fiocruz, Rio de Janeiro, Brazil; ^6^ Laboratório de Pesquisa em Malária, Instituto Oswaldo Cruz (IOC), Fiocruz, Rio de Janeiro, Brazil; ^7^ Escola de Enfermagem Aurora de Afonso Costa, Departamento de Doenças infecciosas e Parasitárias, Universidade Federal Fluminense, Niterói, Brazil; ^8^ Fundação Universidade Regional de Blumenau (FURB), Blumenau, Brazil; ^9^ Centro de Pesquisas Biológicas de Indaial, Indaial, Brazil; ^10^ Programa de conservação do Bugio Ruivo, Perini Business Park, Joinville, Brazil; ^11^ Centro de Primatologia do Rio de Janeiro (CPRJ), Instituto Estadual do Ambiente (INEA), Guapimirim, Brazil; ^12^ Centro Universitário Serra dos Órgãos (Unifeso), Teresópolis, Brazil; ^13^ Fundação de Parques Municipais e Zoobotânica (FPMZB), Belo Horizonte, Brazil; ^14^ Departamento de Análises Clínicas e Toxicológicas, Faculdade de Ciências Farmacêuticas, Universidade de São Paulo, São Paulo, Brazil; ^15^ Center for Global Health and Infectious Diseases Research, College of Public Health, University of South Florida, Tampa, FL, United States; ^16^ Laboratório de Imunoparasitologia, Instituto Oswaldo Cruz (IOC), Fiocruz, Rio de Janeiro, Brazil

**Keywords:** malaria, *Plasmodium*, IgM antibodies, neotropical primates, Atlantic forest, pre-erythrocytic stage antigen, erythrocytic stage antigens

## Abstract

**Introduction:**

Zoonotic transmission is a challenge for the control and elimination of malaria. It has been recorded in the Atlantic Forest, outside the Amazon which is the endemic region in Brazil. However, only very few studies have assessed the antibody response, especially of IgM antibodies, in Neotropical primates (NP). Therefore, in order to contribute to a better understanding of the immune response in different hosts and facilitate the identification of potential reservoirs, in this study, naturally acquired IgM antibody responses against Plasmodium antigens were evaluated, for the first time, in NP from the Atlantic Forest.

**Methods:**

The study was carried out using 154 NP samples from three different areas of the Atlantic Forest. IgM antibodies against peptides of the circumsporozoite protein (CSP) from different *Plasmodium* species and different erythrocytic stage antigens were detected by ELISA.

**Results:**

Fifty-nine percent of NP had IgM antibodies against at least one CSP peptide and 87% against at least one *Plasmodium* vivax erythrocytic stage antigen. Levels of antibodies against PvAMA-1 were the highest compared to the other antigens. All families of NP showed IgM antibodies against CSP peptides, and, most strikingly, against erythrocytic stage antigens. Generalized linear models demonstrated that IgM positivity against PvCSP and PvAMA-1 was associated with PCR-detectable blood-stage malaria infection and the host being free-living. Interestingly, animals with IgM against both PvCSP and PvAMA-1 were 4.7 times more likely to be PCR positive than animals that did not have IgM for these two antigens simultaneously.

**Discussion:**

IgM antibodies against different *Plasmodium* spp. antigens are present in NP from the Atlantic Forest. High seroprevalence and antibody levels against blood-stage antigens were observed, which had a significant association with molecular evidence of infection. IgM antibodies against CSP and AMA-1 may be used as a potential marker for the identification of NP infected with Plasmodium, which are reservoirs of malaria in the Brazilian Atlantic Forest.

## Introduction

Malaria remains a critical public health concern despite all the efforts to control the disease around the world. The World Health Organization estimated 247 million malaria cases and 619,000 deaths in 2021 (World Health Organization, 2022). In Brazil, around 140,000 cases were reported in 2021, of which 83% were caused by *P. vivax*, 17% by *Plasmodium falciparum*, and <1% by *Plasmodium malariae* (SVS/MS, 2022a). More than 99% of malaria cases in Brazil occur in the Amazon region. However, autochthonous cases and sporadic outbreaks of malaria occur outside the Amazonian region and are of great concern because of the high mortality rate associated with such infections (SVS/MS, 2022a). Between 2010 and 2021, more than 8000 cases of malaria were confirmed outside the Amazon region, including those occurring in the Atlantic Forest region along the eastern Atlantic coast of Brazil (SVS/MS, 2022b).

One of the greatest challenges for the control and elimination of malaria in the world is zoonotic transmission. The huge impact of zoonotic transmission of malaria was recently described in South-East Asia, which was caused by *P. knowlesi*, a non-human primate malaria parasite (Cox-Singh, 2012). In Brazil, there are two malaria parasite species that infect Neotropical primates (NP): *Plasmodium simium* and *Plasmodium brasilianum. Plasmodium simium* infects primates of Atelidae, Cebidae, and Pitheciidae families in the Atlantic Forest from south and southeastern Brazil (da Fonseca, 1951; Deane, 1992; de Alvarenga et al., 2015). The primate species most frequently infected with *P. simium* is *Alouatta guariba clamitans* (southern brown howler monkey) (Deane, 1992; de Alvarenga et al., 2015; Abreu et al., 2019). *Plasmodium brasilianum* infects primates of all NP families found in Central America to southern Brazil (Seidelin, 1912; Deane, 1992; Lourenço-de-Oliveira and Deane, 1995; Lalremruata et al., 2015; Alvarenga et al., 2017). *Plasmodium simium* and *P. brasilianum* are similar in morphology, genetics, and immunology to human *Plasmodium* species, *P. vivax*, and *P. malariae*, respectively (Coatney, 1971; Cochrane et al., 1985; Barnwell, 1986; de Arruda et al., 1989; Deane, 1992; Escalante et al., 1995; Fandeur et al., 2000; Tazi and Ayala, 2011). Zoonotic transmission of *P. brasilianum/Plasmodium malariae* between NP and indigenous people from the Yanomami community in the Venezuelan Amazon was confirmed using molecular biology techniques (Lalremruata et al., 2015). In Brazil, an outbreak of zoonotic human malaria caused by *P. simium* was recently reported in the Atlantic Forest region in the state of Rio de Janeiro (Brasil et al., 2017; Mourier et al., 2021).

The immune response to malaria parasites is complex and occurs against distinct antigenic combinations, composed of antigens from each stage of the parasite’s life cycle (Struik and Riley, 2004). The vertebrate host uses different effector mechanisms involving innate and adaptive immunity. It is well-established that in humans, naturally acquired IgG antibody responses are associated with protective clinical immunity to malaria (Cohen et al., 1961; Sabchareon et al., 1991). Although IgM may play a role in malaria immunity, such a role is not well defined as of yet (Pleass et al., 2016; Stone and Lund, 2016). Recently, IgM antibodies were proposed to have a much greater role than just the early response generally stated in classical immunology books (Boonyaratanakornkit and Taylor, 2019). Memory B cells have recently been shown in experimental models to produce specific IgM against *Plasmodium* sp. and dominate the early memory response to recurrent malaria infections, providing evidence of additional mechanisms by which the immune system may control infection (Couper et al., 2005; Bohannon et al., 2016; Krishnamurty et al., 2016). Furthermore, a recent study by Boyle et al. associated higher levels of IgM against *P. falciparum* with a reduced risk of clinical malaria in children (Boyle et al., 2019). Studies evaluating human naturally acquired IgM immune responses against different erythrocytic stage antigens have been conducted in different areas of Brazil (Rodrigues et al., 2005; Oliveira et al., 2006; Riccio et al., 2013, Cassiano et al., 2016; Medeiros et al., 2020; Lima et al., 2022; Schappo et al., 2022). However, only a few studies have looked at the naturally acquired immune response of NP exposed to *Plasmodium* infection, with all such studies focusing on IgG antibodies (Duarte et al., 2006; Yamasaki et al., 2011; Costa et al., 2014; Monteiro et al., 2020; de Assis et al., 2021).

Previously, we performed an immuno-epidemiological study of the IgG antibody responses in NP from epidemiologically distinct malaria transmission areas of the Atlantic Forest in Brazil (de Assis et al., 2021). Here, the naturally acquired IgM antibody levels of NP against both pre-erythrocytic and erythrocytic stage antigens of *Plasmodium* were evaluated. This can contribute to the understanding of the role of IgM in the immune response against malaria in NP and may allow for the identification of NP species with reservoir capacity.

## Materials and methods

### Sera from non-human primates and studied areas

**Figure 1:**
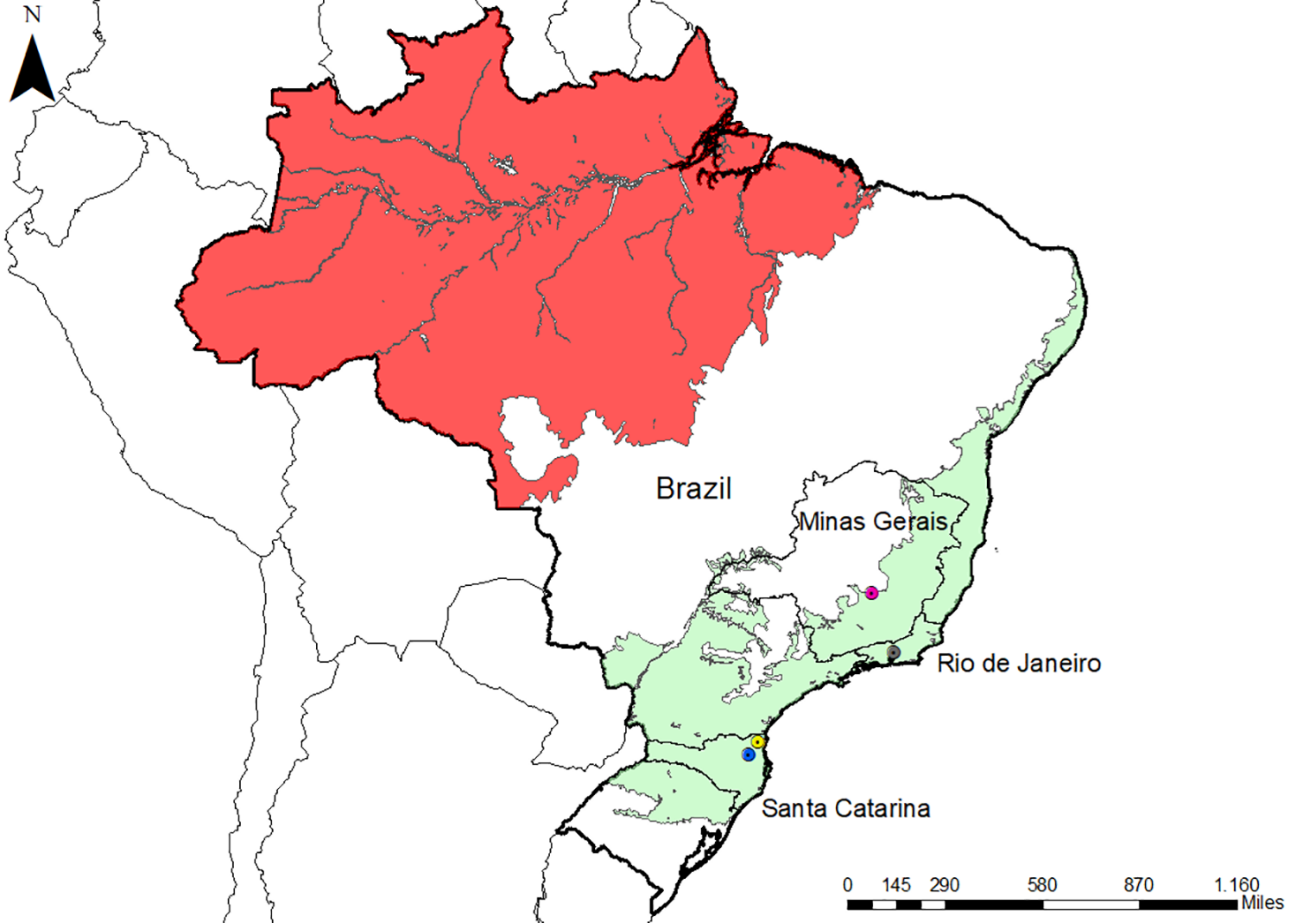
Map of Brazil with the location of the three studied areas. Malaria endemic areas (red) and the Atlantic Forest areas (green) are indicated. The states of the studied areas are highlighted (Rio de Janeiro and Santa Catarina). Icons show three studied municipalities: Indaial (blue), Joinville (yellow), and Guapimirim (grey). The map also highlighted Belo Horizonte (pink) in Minas Gerais state because the negative controls were collected there. The map was made with ArcGIS software.

Serum samples were collected from NP from three areas of the Atlantic Forest in Southeastern and South Brazil (**Table 1** and **Figure 1**). These areas were previously studied by our group and were shown to have distinct epidemiological profiles of malaria transmission (Costa et al., 2014; de Alvarenga et al., 2018; Nunes et al., 2020; de Assis et al., 2021). Samples from a total of 154 NP belonging to all families (Aotidae, Atelidae, Callitrichidae, Cebidae, and Pithecidae) from the biological repository of our laboratory were selected for this study (**Table 1**).

**Table 1:** Characteristics of the Neotropical primates sampled from each studied area.

Characteristic	Studied area (Municipality/State)			Total
	Indaial/SC	Joinville/SC	Guapimirim/RJ	
	*n* = 75	*n* = 39	*n* = 40	*n* = 154
**Sex ratio male:female**	1.27:1	0.95:1	1:1.22	1.05:1
**Ratio Adult: Non-adult** *^a^*	4.35:1	5.5:1	40:0	6.7:1
**Ratio Captive: Free-living**	5.25:1	0:39	40:0	2.01:1
Families of NP				
Atelidae	75 (100%)*^b^*	39 (100%)*^b^*	8 (20%)*^c^*	122 (79.2%)
Aotidae	0	0	1 (2.5%)*^d^*	1 (0.6%)
Callitrichidae	0	0	4 (10%)*^e^*	4 (2.6%)
Cebidae	0	0	24 (60%)*^f^*	24 (15.6%)
Pitheciidae	0	0	3 (7.5%)*^g^*	3 (1.9%)
Positivity by PCR*^h^*	10 (13%)	26 (67%)	13 (32%)	59 (32%)
Positivity by ELISA (IgG)*^i^*				
PvCSP	50 (67%)	39 (100%)	34 (85%)	123 (80%)
Pb/PmCSP	47 (63%)	39 (100%)	35 (87.5%)	121 (76%)
PfCSP	54 (72%)	38 (97%)	34 (85%)	126 (82%)
PvAMA-1	52 (69%)	39 (100%)	5 (12.5%)	96 (62%)
PvEBP-2	52 (69%)	39 (100%)	7 (17.5%)	98 (64%)
PvDBPII	48 (64%)	39 (100%)	7 (17.5%)	94 (61%)

Results are expressed in absolute numbers and percentages in parentheses. ^a^Age was estimated according to Carpenter, 1965. Studied species of the Neotropical primates: ^b^ *Alouatta guariba clamitans, ^c^Alouatta g. clamitans, Alouatta caraya, Ateles paniscus, Brachyteles arachnoides; ^d^Aotus nigriceps; ^e^Mico humeralifer, Leontopithecus chrysomelas, Leontopithecus rosalia, Saguinus midas; ^f^Cebus* sp., *Sapajus robustus, Sapajus xanthosternus; ^g^Cacajao melanocephalus, Callicebus personatus*. ^h^Molecular diagnosis of *Plasmodium* sp. infection was previously performed by our group **(** Costa et al., 2014 **;** de Alvarenga et al., 2015 **;** Alvarenga et al., 2017 **;** de Alvarenga et al., 2018 **;** Nunes et al., 2020). ^i^ Enzyme-linked immunosorbent assay for IgG detection using different antigens of *Plasmodium* was previously performed by our group (de Assis et al., 2021). SC – state of Santa Catarina; RJ – state of Rio de Janeiro.

The NP samples that we studied came from three different locations in the Atlantic Forest: Indaial and Joinville, both in the state of Santa Catarina (SC) in south Brazil, and Guapimirim in the state of Rio de Janeiro (RJ) in southeast Brazil (**Table 1**). Samples from three *Alouatta g. clamitans* from the Fundação de Parques Municipais e Zoobotânica at Belo Horizonte in the state of Minas Gerais (MG), Brazil, outside the Atlantic Forest in a non-transmission malaria area were used as negative controls.

The first group of samples from Indaial comprised 75 plasma samples all taken from brown howler monkeys (*Alouatta guariba clamitans*) caught between 2008 and 2019: 63 from captive monkeys maintained at the Centre for Biological Research of Indaial (CEPESBI), and a further 12 plasma samples taken from free-living NP. In this group, 81% of samples were from adult NP (**Table 1**), and *Plasmodium* infection was previously identified by our group in 13% of these NP by PCR (Costa et al., 2014; de Alvarenga et al., 2018). In our previous study, we reported that 75% of these samples were seropositive for IgG responses against pre-erythrocytic stage antigens (CSP peptides) and 90% of them against different erythrocytic stage antigens (PvAMA-1, PvEBP-2, and PvDBPII) of *P.vivax* (de Assis et al., 2021) (**Table 1**).

The second group of samples was collected in the city of Joinville in an Atlantic Forest conservation area located on private property. These plasma samples were collected from 39 free-living brown howler monkeys between December 2015 and 2017 (for more details about the capture see Nunes et al., 2020). Sampling from adults was prioritized during the captures which made up 85% of the monkeys sampled. *Plasmodium* infection was previously identified in 67% of these selected samples, which were infected with either *P. simium* and/or *P. brasilianum* according to PCR (Nunes et al., 2020). All of these animals showed IgG antibodies against both pre-erythrocytic and erythrocytic stage antigens (de Assis et al., 2021) (**Table 1**).

The third group of samples studied came from the Primate Centre of Rio de Janeiro (CPRJ) in Guapimirim, where serum samples were collected between January 2011 and October 2019 from 40 captive adult monkeys belonging to all NP families (**Table 1**). *Plasmodium* infection was previously identified in 32% of selected samples by PCR (de Alvarenga et al., 2015; Alvarenga et al., 2017). Ninety percent of these animals showed IgG against CSP peptides and 32% against erythrocytic stage antigens (de Assis et al., 2021) (**Table 1**).

All procedures were performed according to Brazilian guidelines and regulations and were approved by the Fiocruz Research Ethical Committee (CEUA license L037/2016) and by the Brazilian Ministry of Environment (SISBIO numbers 43375-4, 43375-6, 54707-137362-2, 52472-1, and 28953-1).

### Peptides and recombinant *Plasmodium* antigens

#### Pre-erythrocytic stage antigens

Synthetic peptides corresponding to the repeated immunodominant epitope of the major pre-erythrocytic stage antigen, named the Circumsporozoite Protein (CSP), were used for the detection of immunoglobulin M (IgM) antibodies. Three variants of *P. vivax* CSP (PvCSP) were used: the classic VK210 (Pvc) DGQPAGDRAAGQPAG-(DRADGQPAG)_2_, VK247 (Pvk) (ANGAGNQPG)_3_-ANGAGN, and *P. vivax-*like (Pvl) (APGANQEGGAA)_3_. Additionally, CSP peptides from *P. falciparum* (NANP)_8_ (PfCSP) and *P. malariae/P. brasilianum* (GNAA)_2_-GNDA(GNAA)_4_ were utilized. The CSP repeat sequences of *P. brasilianum* and *P. malariae* are identical, and because of that, they were referred to as Pb/PmCSP (Guimarães et al., 2012; Pereira et al., 2018).

#### *Plasmodium* spp. erythrocytic stage antigens

As previously described, the following erythrocytic stage antigens were produced as recombinant proteins. Recombinant DBPII proteins included amino acids 243–573 of Duffy Binding Protein (DBP) from the Sal-1 reference strain, and recombinant EBP2 included amino acids 159–485 of the Erythrocyte Binding Protein 2 from C127 Cambodian isolate. These two recombinant proteins were expressed as 39 kDa 6xHis-tag fusion protein in *Escherichia coli* (Fang et al., 1991; Cerávolo et al., 2005; Ntumngia et al., 2012; Ntumngia and Adams, 2012; Ntumngia et al., 2016). The ectodomain of *P. vivax* apical Membrane Antigen 1 (PvAMA-1), including aminoacids 43 to 487, was produced as a recombinant protein with 6xHis-tag in the eukaryotic expression system (Cunha et al., 2014; Vicentin et al., 2014). The Merozoite Surface Protein-1 protein was additionally used to better characterize the NP responses based on previous studies that indicated that it is highly immunogenic under natural conditions of human exposure (Soares et al., 1997; Riccio et al., 2013; Uplekar et al., 2017; Kale et al., 2019). The 19-kDa C-terminal fragment of the MSP-1 from *P. vivax*, *P. falciparum*, and *P. malariae* (MSP1-19), which represents amino acids 1616–1704, was expressed as a 6xHis tag fusion protein in *Pichia pastoris* (Soares and Rodrigues, 2002).

### Immunoglobulin M detection assay

The enzyme-linked immunosorbent assay (ELISA) for the detection of antibodies against *Plasmodium* antigens was performed, as previously described (Duarte et al., 2006; Pereira et al., 2018; de Assis et al., 2021) with modifications. Briefly, all CSP synthetic peptides were used at 10 μg/mL to coat the plates, while recombinant proteins were used at a final concentration of 1 µg/mL (PvMSP1_19_), 1.5 µg/mL (PvEBP-2 and PvAMA-1), 3 µg/mL (PvDBPII), and 5 µg/mL (PmMSP1_19_ and PfMSP1_19_). NP serum or plasma samples were diluted at 1:50. Anti-human IgM-peroxidase-conjugated antibody (Jackson Immunoresearch) was used as a secondary antibody at 1:2500 dilution for CSP peptides or 1:5000 for recombinant proteins. In each well was added 100 μL of the 3,3′,5,5′-Tetramethylbenzidine (TMB) single solution (Life Technologies). Reaction development was interrupted by adding 25 μL of 2N HCl in each well. Antibodies were detected using a microplate reader (Spectra Max 340PC 384, Molecular Devices) at an optical density (OD) of 450 nm. Results were expressed as seroprevalence, i.e., the percentage of positives, and antibody levels were expressed as the reactivity index (RI), which was calculated by dividing the mean OD values of tested samples by the mean plus three standard deviations (SDs) of the negative control samples. Samples used as negatives were taken from three *Alouatta g. clamitans* from Belo Horizonte in the state of Minas Gerais, Brazil, a non-transmission malaria area. This species was selected as it corresponded to 77% of the non-control animals studied here. These samples were negative in the *Plasmodium* PCR protocols tested (Snounou et al., 1993; de Alvarenga et al., 2018). Samples with an RI greater than one were considered positive. For comparisons with immune responses against erythrocytic stage antigens, samples were considered positive for PvCSP when they showed an RI >1 for any of the three CSP repeats (VK210, VK247, or *P. vivax*-like).

### Statistical analysis

Analyses were done using GraphPad Prism version 8.0 (GraphPad Software, Inc., San Diego, CA, USA) and R version 4.0.2 (Vienna, Austria) (R Core Team, 2020). For each area of study, the distribution of the data for each of the parameters was determined by the Kolmogorov-Smirnov and Shapiro-Wilk tests. Differences in the proportion of the seropositive were evaluated using either Fisher’s exact test or the Chi-square test (*_X_* ^2^), according to the data distribution. The differences between either the medians or means of the reactivity index (RI) of two groups were verified using either the *t*-test or the Mann-Whitney *U* test, respectively, according to the data distribution. The RI comparisons between more than two groups were performed using either Analysis of Variance (ANOVA) or the Kruskal-Wallis test, followed by Tukey’s or Dunn’s post-hoc tests, respectively, according to the data distribution. For analysis of the overall data set, i.e., the data from all three groups combined, the association between categorical variables (age, sex, whether free-living or captive, and the occurrence of active infection – as determined by PCR) and seroprevalence and IgM titers was assessed using either the Chi-square test (*_X_* ^2^) or Fisher’s exact test, and the *t*-test or Mann-Whitney *U* test, respectively. Heatmaps for antibody levels against *Plasmodium* antigens were created using the R package *ComplexHeatmap*, which showed the hierarchical clustering with families fixed (Gu et al., 2016). The intersections of responders to PvCSP and *P. vivax* erythrocytic stage antigens were plotted using the “*UpSetR*” R package (v1.4.0) (Conway et al., 2017). Analyses of correlation between antibody levels of responders (i.e., those individuals classified as seropositive) were assessed using Spearman’s rank correlation coefficient. For analyses of the categorical variables described above, the odds ratios for individuals to have antibodies against given antigens of the various *Plasmodium* spp. tested, generalized linear models (Gamma regression model and logistic regression), and the Gamma-binomial mixture model were used. Variables with *P*-values < = 0.20 in the likelihood ratio test went on to step 2, where all were jointly adjusted. From that moment on, all the variables that passed from the previous step were adjusted together. The significance of each was analyzed separately using the likelihood ratio test. Here, we compared whether there was a significant difference between the model without and with the variable in question. A model was fitted with all significant variables from the previous step. The process was repeated until only significant variables remained. In all analyses, a significance level of 5% was considered, i.e., values of *P*< 0.05.

## Results

### IgM responses against pre-erythrocytic stage CSP peptides

**Figure 2:**
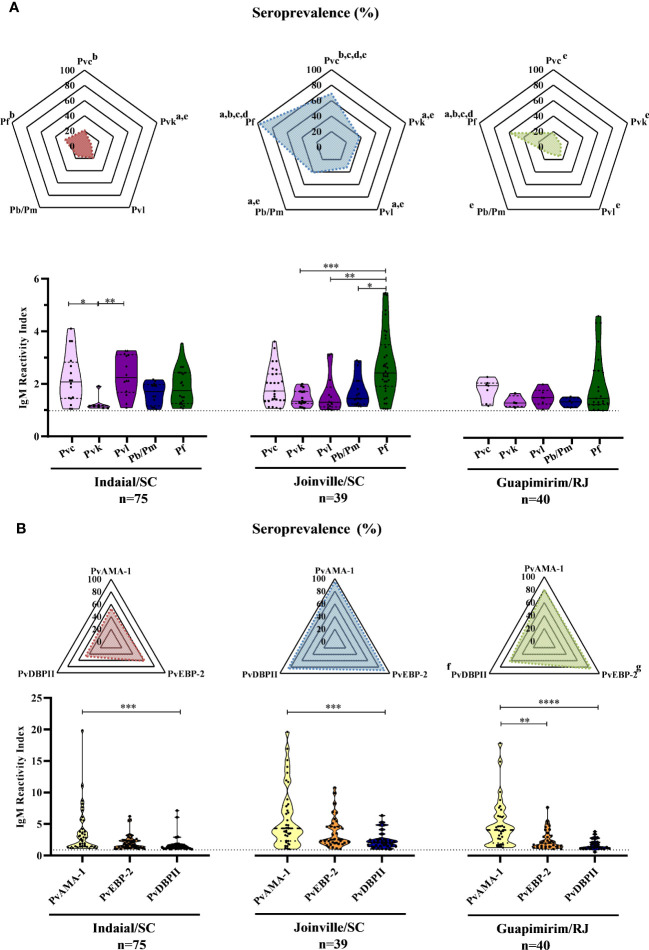
Seroprevalence and levels of IgM antibodies against *Plasmodium* antigens in Neotropical primates. Seroprevalence and reactivity index (RI) of IgM antibodies against **(A)** circumsporozoite protein (CSP) peptides and **(B)** *Plasmodium vivax* erythrocytic stage antigens of NP from Indaial and Joinville in the state of Santa Catarina and Guapimirim in the state of Rio de Janeiro. RI>1 was considered positive (dotted line in violin plots). The radar chart indicates the percentage of NP with RI>1. In the violin plots, the data shown are the individual RI values for each sample (dots) with their median and the interquartile range. Pvc, variant classic VK210 of the *P. vivax* CSP (lilac); Pvk, variant VK247 of the *P. vivax* CSP (pink); Pvl, variant *P. vivax-*like of the *P. vivax* CSP (purple); Pb/Pm, CSP of *P. brasilianum/P. malariae* (dark blue); Pf, CSP of *P. falciparum* (dark green); PvAMA-1, *P. vivax* Apical Membrane Antigen-1 (yellow); PvEBP-2, *P. vivax* Erythrocyte Binding Protein 2 (Orange); PvDBPII*, P. vivax* Duffy Binding Protein region II (blue). At the bottom of each graph is shown the numbers (*n*) of NP included from each area. Statistically significant differences are indicated by lowercase letters for radar plots (a – compared to Pvc (*P*< 0.05); b – compared to Pvk (*P*< 0.05); c – compared to Pvl (*P*< 0.01); d – compare to Pb/Pm (*P*< 0.05); e – compared to Pf (*P*< 0.01); f – compared to PvEBP-2 (*P*< 0.05); g – compared to PvDBPII (*P*< 0.05), and asterisks for violin plots (*P> 0.05, **P> 0.01, ***P> 0.001, and ****P<0.0001).

Overall, 59% (91/154) of the NP showed an IgM response against at least one of the five CSP peptides assayed (**Supplementary Figure 1A**). The responses against at least one of the PvCSP peptides (Pvc, Pvk, and Pvl) ranged from 37% (Guapimirim/SC) to 87% (Joinville/SC). Twenty-seven percent (Indaial/SC) and up to 97% (Joinville/SC) of the NP also showed IgM responses against PfCSP. From 10% (Guapimirim/RJ) to 41% (Joinville/SC) showed IgM antibodies against Pb/PmCSP (**Figure 2A** top). The frequency of seropositive NP was significantly highest against PfCSP and *P. vivax* “classic” VK210 (Pvc) in all three areas (*P<* 0.05) (**Figure 2A** top).

In Indaial/SC, antibody levels were significantly higher against Pvc and Pvl than against the Pvk peptide (*P<* 0.05). In Joinville/SC, antibody levels were significantly higher against PfCSP than against PvkCSP, PvlCSP, and Pb/PmCSP peptides (*P<* 0.05). The levels of antibodies were relatively low and without any statistically significant differences among all CSP peptides in Guapimirim/RJ (**Figure 2A** bottom).

### IgM responses against *Plasmodium vivax* erythrocytic stage antigens

Overall, significant levels of IgM were identified in 87% (134/154) of the NP studied against at least one of the three *P. vivax* erythrocytic stage antigens assayed (PvAMA-1, PvEBP-2, and PvDBPII) (**Supplementary Figure 1B**). The seroprevalence for at least one of the three erythrocytic stage antigens was higher for Joinville/SC and Guapimirim/RJ (both 95%) than Indaial/SC (76%). The frequency of positive NP was significantly higher against PvEBP-2 than against the other antigens only in Guapimirim/RJ (*P<* 0.05) (**Figure 2B** top). Antibody titers against PvAMA-1 were higher than PvDBPII in all three of the areas studied. In Guapimirim/RJ, the levels of anti-PvAMA-1 IgM were also significantly higher than against PvEBP-2 (*P<* 0.05) (**Figure 2B** bottom).

### Association between the seroprevalence and antibody levels against PvCSP and erythrocytic stage antigens of *P. vivax*

**Figure 3:**
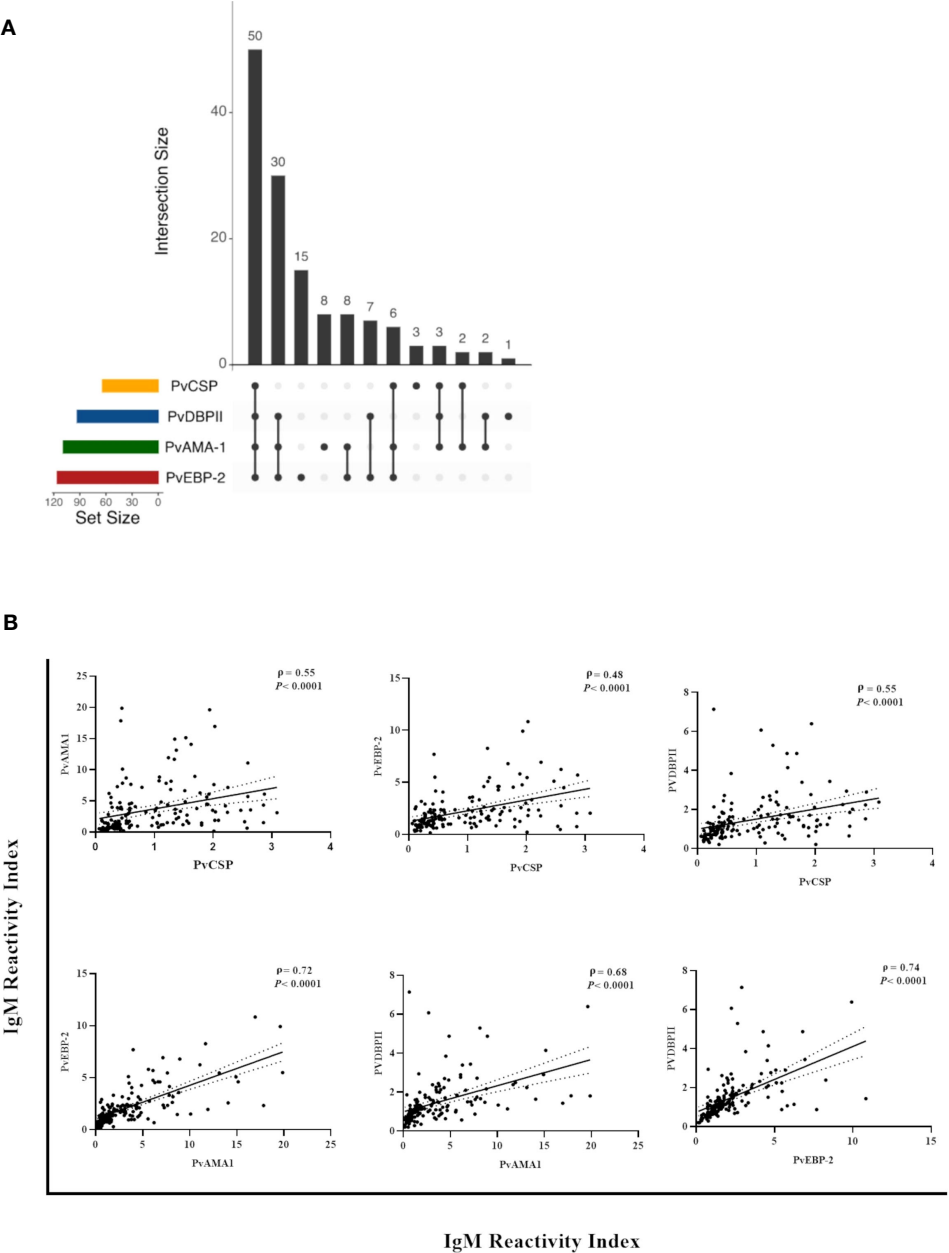
Correlation between IgM responses against pre-erythrocytic and erythrocytic stage antigens of *P. vivax* among Neotropical primates. **(A)** UpSetR plot of the number of NP with IgM against PvCSP and/or *P. vivax* erythrocytic stage antigens. **(B)** Significant correlations between the reactivity index of IgM against PvCSP and *P. vivax* erythrocytic stage antigens and between the different erythrocytic stage antigens from this *Plasmodium* species. Data shown are the value of ρ (Spearman’s rank coefficient), its significance as indicated by the *P*-value, and the dotted lines indicate the 95% confidence intervals. PvCSP, *P. vivax* CSP repeats (VK210, VK247, and/or *P.vivax*-like variants); PvAMA-1, *P. vivax* Apical Membrane Antigen 1; PvEBP-2, *P. vivax* Erythrocyte Binding Protein 2; PvDBPII, *P. vivax* Duffy Binding Protein region II.

Thirty-seven percent (50/135) of the NP showed IgM antibodies against all of the studied antigens (i.e., all three pre-erythrocytic and all three erythrocytic stage antigens from *P. vivax*), while 45% (61/135) of the NP showed IgM against all three variants of PvCSP and at least one erythrocytic stage antigen (either PvAMA-1, PvEBP-2, and/or PvDBPII), and 53% (71/135) of the individuals showed IgM against at least one erythrocytic stage antigen but not against CSP (**Figure 3A**). Significant positive correlations ranging from moderate to strong were identified (*P<* 0.001) between antibody levels against PvCSP and PvAMA-1 (ρ = 0.55), PvCSP and PvEBP-2 (ρ = 0.48), PvCSP and PvDBPII (ρ = 0.55), PvAMA-1 and PvEBP-2 (ρ = 0.72), PvAMA-1 and PvDBPII (ρ = 0.68), and PvEBP-2 and PvDBPII (ρ = 0.74) (**Figure 3B**). These comparisons were also performed when classifying the NP according to PCR positivity for malaria infection; however, the previously described significant correlations were not altered according to this parameter (data not shown).

### Variables potentially associated with seroprevalence and IgM levels against CSP and erythrocytic stage antigens

**Figure 4:**
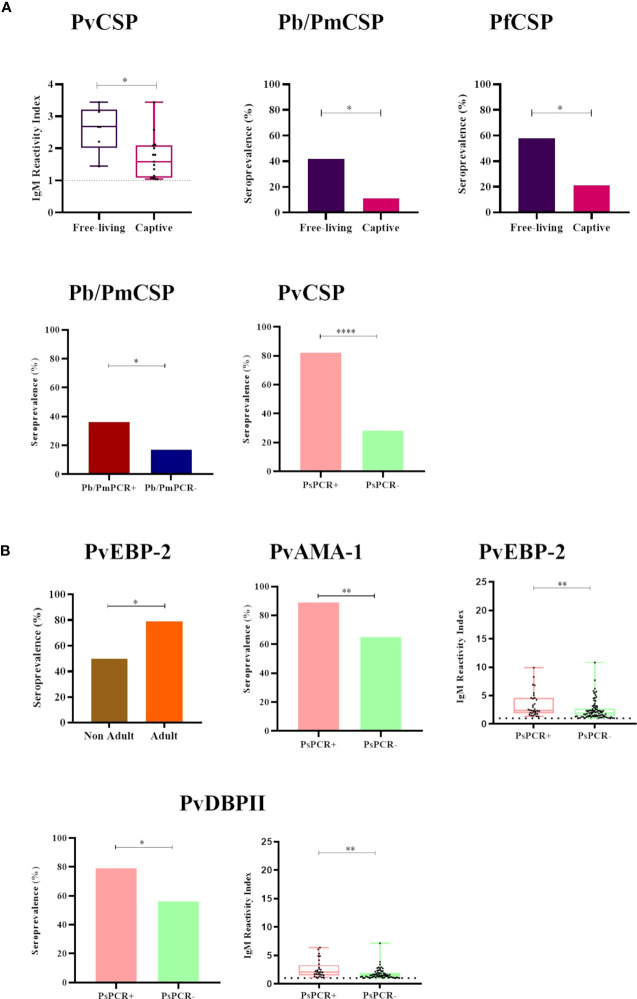
Statistically significant differences in the seroprevalence and levels of IgM antibodies against *Plasmodium* antigens according to different sampling parameters. **(A)** Comparison of IgM seroprevalence and levels against CSP peptides of *Plasmodium* sp. between (i) free-living (purple) and captive (pink) individuals, and (ii) malaria parasite PCR-positive (PCR+, red for *P. brasilianum/P. malariae* and salmon for *P. simium*) and PCR-negative samples (PCR-, blue for *P. brasilianum/P. malariae* and light green for *P. simium*). **(B)** Comparison of IgM seroprevalence and levels against *P. vivax* erythrocytic stage antigens between (i) non-adult (brown) and adult (orange) individuals and (ii) malaria parasite PCR-positive (PCR+, salmon for *P. simium*) or PCR-negative (PCR-, light green for *P. simium*). Samples with an RI>1 were considered seropositive (dotted line). The filled bars indicate the percentage of NP with an RI> 1. The data shown on the graphs of IgM levels are the individual RI values for each sample (dots) with their median and the interquartile range (boxes). The ages of NP were estimated according to Carpenter (1965) and accordingly categorized into non-adult and adult. PvCSP, *P. vivax* CSP variants (VK210, Vk247, and *P.vivax*-like); Pb/PmCS, CSP repeats of *P. brasilianum/P. malariae*; PfCSP, CSP repeats of *P. falciparum*; PvEBP-2, *P. vivax* Erythrocyte Binding Protein 2; PvAMA-1, *P. vivax* Apical Membrane Antigen 1; PvDBPII, domain II of the *P. vivax* Duffy Binding Protein. Statistically significant differences are indicated by asterisks (*P> 0.05, **P> 0.01, and ****P> 0.0001). The free-living versus captive comparison was performed only for the group of NP from Indaial/SC.

Free-living NP showed both significantly higher IgM titers against PvCSP and a higher seroprevalence against Pb/PmCSP and PfCSP when compared to captive animals (*P*<0.05) (**Figure 4A**). All comparisons of free-living and captive NP are shown in **Supplementary Figure 2**. Adult animals also had significantly higher seroprevalence against PvEBP-2 than non-adults NP (*P<*0.05) (**Figure 4B** and **Supplementary Figure 3**). Seroprevalences and IgM titers were similar between the sexes for the CSP peptides and erythrocytic stage antigens (*P*>0.05) (**Supplementary Figure 4**).

Among NP PCR-positive for *P. brasilianum/P. malariae*, the seroprevalence against Pb/PmCSP was higher than in PCR-negative monkeys for this infection (seroprevalences: 36% and 18%, respectively, *P>*0.05), while IgM titers were similar (**Figure 4A** and **Supplementary** **Figure 5**). Frequencies of seropositivity against PvCSP, PvAMA-1, and PvDBPII were higher in NP infected with *P. simium* (PCR- positive for this malaria parasite) compared to NP non-infected with *P. simium* (PCR- negative for *P. simium*) (*P*<0.05, Chi-square test) (**Figure 4**). NP PCR-positive for *P. simium* also showed higher levels of IgM against the PvEBP-2 and PvDBPII than PCR-negative individuals (*P>* 0.05) (**Figure 4** and **Supplementary Figure 5**).

**Figure 5:**
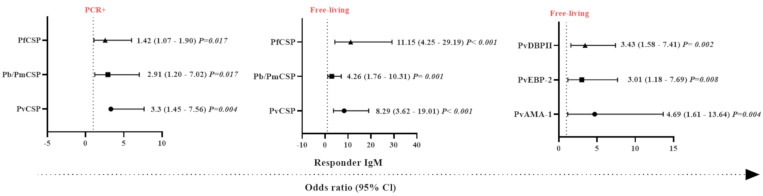
Odds ratios for the IgM antibody responses of Neotropical primates against different *Plasmodium* antigens according to different sampling parameters. Comparison of the odds ratios for seroprevalence of IgM against various CSP peptides from different *Plasmodium* species and *Plasmodium vivax* erythrocytic stage antigens according to malaria parasite PCR positivity and whether sampled individuals were either free-living or captive. PvCSP, *P. vivax* CSP repeat variants (VK210, VK247, and *P. vivax-like*); Pb/PmCSP, CSP repeat of *P. brasilianum/P. malariae*; PfCSP, CSP repeat of *P. falciparum*; PvAMA-1, *P. vivax* Apical Membrane Antigen 1; PvEBP-2, *P. vivax* Erythrocyte Binding Protein 2; PvDBPII, domain II of the *P. vivax* Duffy Binding Protein. The data shown are only the significant values for the odds ratios (logistic Regression, *P*<0.05). The free-living versus captive comparison was performed only for the group of NP from Indaial/SC.

### Generalized linear models and the identification of factors associated with IgM response against *Plasmodium* spp. antigens

In order to identify which variables could explain the occurrence of IgM antibodies against *Plasmodium* spp., generalized linear models (gamma regression model, logistic regression, and the Gamma-binomial mixture model) were used. With respect to the response against the pre-erythrocytic stage protein, CSP, individuals that were PCR-positive for malaria infection showed a higher chance of having IgM antibodies against PfCSP, Pb/PmCSP, and PvCSP compared to PCR-negative NP (*P <*0.05, Logistic Regression) (**Figure 5**). The highest probability of being seropositive for IgM was observed against PvCSP (3.3 times more likely). Free-living NP also showed a higher chance of having IgM against PfCSP, Pb/PmCSP, and PvCSP. The highest odds ratio for being seropositive with IgM antibodies was observed against PfCSP, which was 11 times more likely. In relation to erythrocytic stage proteins, free-living individuals showed a higher chance of an IgM response against all three antigens. The highest odds ratio was almost five times greater than for captive NP, which was for IgM against PvAMA-1 (*P<*0.008) (**Figure 5**).

Individually, a set of variables (PCR-positive and free-living) was identified that determined the chance of NP having IgM against erythrocytic stage proteins. PCR positive (3.66 times, CI = 1.27-10.51 and *P =* 0.016), adult (4.29 times, CI = 1.39-13.25 and *P =* 0.011), and free-living (4.69 times, CI = 1.61 – 13.64 and *P =* 0.004) individuals were significantly more likely to respond against PvAMA-1 compared to PCR-negative, non-adult, and captive individuals, respectively (*P*<0.05). Interestingly, we observed that animals that had IgM against PvCSP and PvAMA-1 were 4.7 times more likely to be PCR-positive, compared to those individuals that did not respond to these proteins (*P*<0.05) (**Supplementary Figure 6**).

### Association between antibody levels against pre-erythrocytic stage peptides and Merozoite Surface Protein 1_19_ antigens of *Plasmodium* spp.

**Figure 6:**
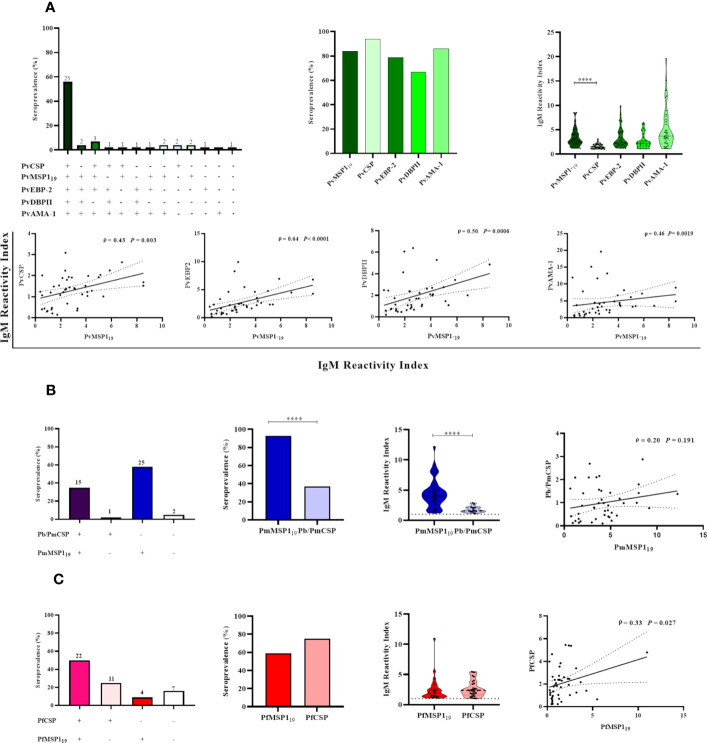
Association between active malaria infection and IgM antibody responses against different *Plasmodium* antigens in Neotropical primates. IgM seroprevalence and reactivity indices (RI>1) of NP against individual malaria parasite antigens, and different combinations of those antigens: *P. vivax* antigens **(A)**, *P. brasilianum/P. malariae* antigens **(B)**, and *P. falciparum* antigens **(C)**. In the leftmost graphs, the absolute number of NP is indicated above the bars. In the graphs showing the IgM reactivity index, the data shown are the individual RI values for each sample (dots), and their median and the interquartile range (violin plot). In the graphs showing the correlations, the data shown are the value of ρ (Spearman’s rank correlation coefficient), with the *P*-value, and the dotted lines indicate the 95% confidence intervals. PvCSP, *P. vivax* CSP repeats (VK210, VK247, and/or *P.vivax*-like); Pb/PmCSP, CSP repeats of *P. brasilianum/P. malariae;* PfCSP, CSP repeats of *P. falciparum*; PvMSP1_19_, fragment of 19kDa of the *P. vivax* Merozoite surface protein 1; PmMSP1_19:_ fragment of 19kDa of the *P. malariae* Merozoite surface protein 1; PfMSP1_19:_ fragment of 19kDa of the *P. falciparum* Merozoite surface protein 1; PvEBP-2, *P. vivax* Erythrocyte Binding Protein 2; PvDBPII, domain II of the *P. vivax* Duffy Binding Protein; PvAMA-1, *P. vivax* Apical Membrane Antigen 1. Statistically significant differences are indicated by asterisks (****P< 0.0001).

After observing an association between IgM response and PCR-detectable blood stage infection, the MSP1_19_ protein was used to further characterize the NP IgM antibody responses. Due to the limited amount of recombinant MSP1_19_ of *P. vivax*, *P. malariae*, and *P. falciparum* available, the presence of IgM antibodies against these antigens was evaluated only in animals with PCR-detectable *Plasmodium* infection (n= 43). Overall, 77% (33/43) of the selected NP samples showed IgM responses against PvMSP_19,_ with 58% (25/43) having IgM antibodies to all *P. vivax* antigens tested. Twenty-three percent (10/43) showed IgM antibodies against one or up to three erythrocytic stage antigens, and 2% (1/43) did not respond against any of the studied antigens. Seroprevalence and antibody titers against PvMSP1_19_ and the other erythrocytic stage *P. vivax* antigens studied were similar. However, there was a statistically significant difference only between PvMSP1_19_ and PvCSP (*P<* 0.05). A significantly weak to moderate positive correlation was also identified between antibody levels against PvMSP1_19_ and other antigens (*P<* 0.003) (**Figure 6A**).

Ninety-three percent of individuals (40/43) showed IgM against PmMSP1_19_, 35% (15/43) showed IgM against Pb/PmCSP and PmMSP1_19_, and 5% (2/43) did not respond against any Pb/Pm antigen. The frequency of responders and their level of antibody against PmMSP1_19_ were higher than against Pb/PmCSP (*P* < 0.001). No correlation was observed between IgM levels against both Pb/Pm antigens (**Figure 6B**).

Sixty percent (26/43) of NP showed IgM against PfMSP1_19_, 50% (22/43) showed IgM against the PfCSP and PfMSP1_19_, and 16% (7/43) did not respond against any one of these antigens. Seroprevalences and antibody titers were similar against both of these antigens (*P>*0.05). A significant weak positive correlation was identified between antibody levels against PfCSP and PfMSP1_19_ (ρ = 0.33 and *P*= 0.027) (**Figure 6C**).

### IgM responses against *Plasmodium* spp. antigens among Neotropical primate families

**Figure 7:**
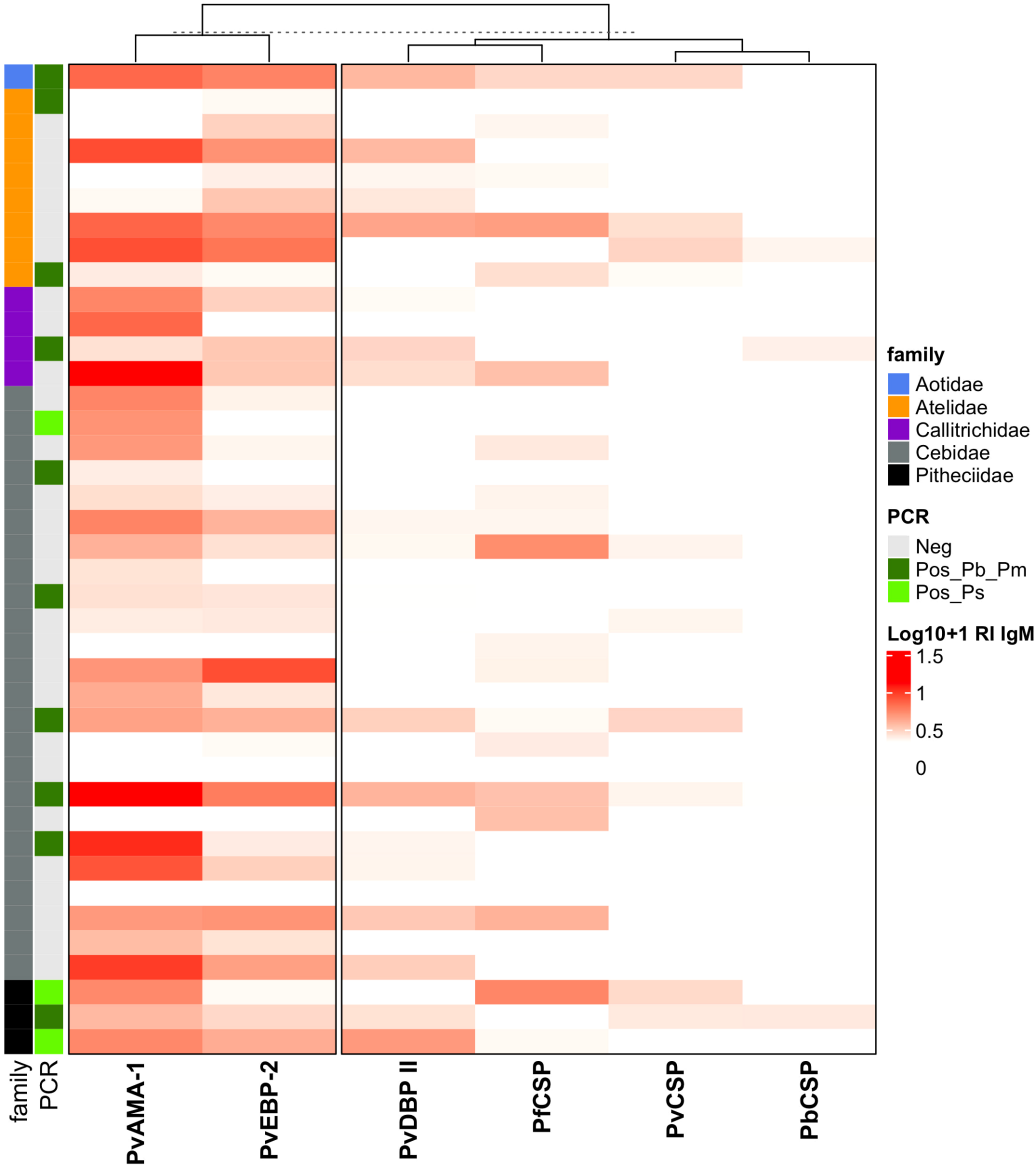
Heatmap of IgM antibody levels against *Plasmodium* antigens among different families of Neotropical primates. IgM antibody levels against CSP repeats from different *Plasmodium* species and *P. vivax* erythrocytic stage antigens among NP families from Guapimirim/RJ are indicated in different intensities of red. The IgM antibody response was expressed as Log10 (Reactivity Index +1). PvCSP, *P. vivax* CSP repeats (VK210, Vk247, and/or *P.vivax*-like); Pb/PmCS, CSP repeats of *P. brasilianum/P. malariae*; PfCSP, CSP repeats of *P. falciparum*; PvAMA-1, *P. vivax* Apical Membrane Antigen 1; PvEBP-2, *P. vivax* Erythrocyte Binding Protein 2; PvDBPII, domain II of the *P. vivax* Duffy Binding Protein. Different NP families are indicated by the colors in the first column, and PCR results for *Plasmodium* are indicated in the second, light green: PCR-positive for *P. simium* (de Alvarenga et al., 2018); dark green: PCR-positive for *P. brasilianum/P. malariae* (Snounou et al., 1993); and grey: PCR-negative for *Plasmodium.* The PCR results shown in this figure were previously published by our group (Costa et al., 2014; de Alvarenga et al., 2015; Alvarenga et al., 2017; de Alvarenga et al., 2018; Nunes et al., 2020).

The analyses of IgM responses among different NP families were performed using samples only from Guapimirim/RJ because the other areas had species only from the Atelidae family (**Table 1**). A unique specimen from the Aotidae family showed low (RI = 1-2) or medium (2<RI<4) levels of IgM antibodies against the three CSP peptides and against erythrocytic stage antigens. Most animals from the Atelidae family had medium to high (RI>=4) levels of antibodies against erythrocytic stage antigens. Sixty-two percent of NP in this family also showed IgM against at least one of the three CSP peptides (Pv, Pb/Pm, and Pf), and 100% showed antibodies against at least one erythrocytic stage antigens (**Figure 7**). Seventy-five percent of NP from the Callitrichidae family had low to medium levels of antibodies against all three CSP peptides, and all individuals from this family showed medium to high levels of antibodies against erythrocytic stage antigens (**Figure 7**). A similar response profile was observed for the animals of the Cebidae family, with 58% of individuals showing low levels of antibodies against at least one CSP peptide, and 92% of NP from this family having IgM against at least one of the erythrocytic stage antigens. All specimens of the Pitheciidae family had low to medium levels of IgM against, at least, one CSP peptide and one erythrocytic stage antigen, generally at high levels (**Figure 7**).

## Discussion

In this study, the profile of acquired IgM antibody immune responses against both pre-erythrocytic and erythrocytic stage antigens of *Plasmodium* was evaluated in NP from different Atlantic Forest areas of Brazil for the first time. The presence of IgM against the major pre-erythrocytic stage antigen (CSP) was used here as a potential marker of exposure to infected mosquito bites.

The frequency of NP showing IgM against CSP peptides was highly variable among the different CSP peptides of different *Plasmodium* species, and the geographic areas were tested (ranging from 10% to 97%). Conversely, the IgM responses were very high for every erythrocytic stage antigen in all studied areas. IgM antibody levels were higher against the PvCSP variant VK210, which is the most common *P. vivax* CSP variant in humans, and NP, which is one of the two variants described for *P. simium* (Goldman et al., 1993; Oliveira-Ferreira et al., 2004; Duarte et al., 2006; Yamasaki et al., 2011; Santos et al., 2019; de Assis et al., 2021). Similar to the IgM response against Pb/PmCSP identified here, IgG antibodies against this peptide were also previously detected among NP from the Atlantic Forest in Brazil by our group and others (Duarte et al., 2006; Yamasaki et al., 2011; de Assis et al., 2021). The high frequency of NP displaying IgM antibodies against the *P. falciparum* CSP, as observed here, also paralleled the previously reported IgG response against this protein in these animals and other NP from different Brazilian states (Duarte et al., 2006; Yamasaki et al., 2011; de Assis et al., 2021). The positive correlation observed in IgM levels against all the studied antigens for each *Plasmodium* species suggests that there is cross-reactivity in the immune response against CSP of different *Plasmodium* species, as previously demonstrated (Hope et al., 1984; Hall et al., 2019).

Free-living animals showed a higher frequency of IgM antibodies against Pb/PmCSP and PfCSP and higher levels of IgM against PvCSP compared to captive animals. These findings may be due to the closer proximity between free-living animals and infected mosquitoes. Similar results were previously observed by our group for IgG antibodies in the same individuals (de Assis et al., 2021). Overall, these data suggest that the seroprevalences of IgM and IgG for *Plasmodium* CSP are good markers of exposure to the transmission of malaria.

Additionally, the detection of antibodies against erythrocytic stage *Plasmodium* antigens was evaluated to characterize immune responses in individuals with established blood-stage *Plasmodium* infections. Recombinant proteins of *P. vivax* blood-stage surface proteins, which are involved in parasite-host interactions and are leading vaccine candidates, were used to evaluate the IgM response of NP from the Atlantic Forest due to the similarity between *P. vivax* and *P. simium*. Here, the highest IgM antibody levels were observed against PvAMA-1 in three studied areas, and high seroprevalences against PvAMA-1 and PvEBP-2 were also detected in some areas. This is the first study to evaluate IgM antibodies against PvEBP-2 in non-human primates. Recently, PvEBP-2 has been suggested as a novel ligand for a potential alternative invasion pathway of Duffy-positive reticulocytes (Hester et al., 2013; Ntumngia et al., 2016). A high seroprevalence of anti-PvEBP-2 IgG was shown in humans from Papua New Guinea, and also for the same NP individuals in our previous study (França et al., 2017; He et al., 2019; de Assis et al., 2021). An IgM response against PvAMA-1 was also previously shown in human individuals recently exposed to *P. vivax* malaria in Brazil (rate of 48.5% responders) (Rodrigues et al., 2005). The low frequency of IgM against PvDBPII was shown here in all three of the studied areas. Medeiros et al. detected anti-PvDBPII IgM in approximately 40% of long-term *P. vivax*-exposed humans in the Amazon (Medeiros et al., 2020). The low immunogenicity of DBPII might be due to the fact that this antigen is only exposed by the merozoite at the time of the reticulocyte invasion, resulting in a short time of contact with the immune system of the host (Adams et al., 1990). IgG antibodies against PvAMA-1 and PvDBPII were previously detected by our group in the same NP individuals studied here (Costa et al., 2014; de Assis et al., 2021).

Furthermore, in order to better characterize the IgM response of NP with established PCR-detectable malaria infection, we used highly immunogenic antigens, the well-characterized MSP1_19_ of *P. vivax*, *P. falciparum*, and *P. malariae* (Soares et al., 1997; Riccio et al., 2013; Uplekar et al., 2017; Kale et al., 2019). A high seroprevalence was observed for both PmMSP1_19_ and PvMSP1_1_, and antibody levels were higher for these antigens compared to the other antigens from the same *Plasmodium* species. These findings confirm the high immunogenicity of MSP1_19_, which is already well-known for humans (Soares et al., 1997; Riccio et al., 2013; Pires et al., 2018; Kale et al., 2019), and NP in areas with circulation of both *P. simium* and *P. brasilianum/P.malariae* of the Atlantic Forest (Duarte et al., 2006; Yamasaki et al., 2011; Monteiro et al., 2020). A high seroprevalence of IgG against PvMSP1_19_ was also reported in NP from the Atlantic Forest in the states of São Paulo and Santa Catarina (Yamasaki et al., 2011; Costa et al., 2014; Monteiro et al., 2020). Overall, these findings corroborate the hypothesis of high circulation of *Plasmodium* spp. between NP in the Atlantic Forest in Brazil (Deane, 1992; de Pina-Costa et al., 2014; Brasil et al., 2017). The significant positive correlations between the IgM responses against pre-erythrocytic and erythrocytic stage antigens found here have also been described in human and non-human primates (França et al., 2017; He et al., 2019; de Assis et al., 2021).

IgM antibodies against PfMSP1_19_ had a lower frequency of responders (60%) compared to MSP1_19_ specific for other *Plasmodium* species, while antibody levels were similar to other Pf antigens (PfCSP). A similar frequency of individuals showing IgG against PfMSP1_19_ (approximately 50%) was reported in human populations in the Amazon region (Braga et al., 2002; Ladeia-Andrade et al., 2007). The IgG seroprevalence against *P. falciparum* antigens in NP and humans and *P. falciparum* infection in humans and mosquitoes have been described in the Atlantic Forest areas of other states in Brazil (Duarte et al., 2006; Cerutti et al., 2007; Marrelli et al., 2007; Yamasaki et al., 2011; Maselli et al., 2014; Laporta et al., 2015; Monteiro et al., 2020). However, NP infection by this species of *Plasmodium* has only been rarely reported in the Atlantic Forest areas (Duarte et al., 2008; Bueno, 2012). Moreover, these findings reinforce the need to investigate the circulation of this *Plasmodium* species or *P. falciparum*-like parasites in this area (Sallum et al., 2014).

Adult NP showed a higher seroprevalence against PvEBP-2 than non-adult animals, suggesting that the IgM response depends on repetitive cycles of erythrocytic stage infection. A primary *P. vivax* infection was not sufficient to induce significant IgM and IgG antibodies to EBP-2 in individuals of a malaria outbreak in the state of Minas Gerais state in Brazil, which is a non-endemic area of malaria transmission (Lima et al., 2022). IgG responses against different erythrocytic stage antigens increase with age due to repeated exposure to *Plasmodium* sp., mainly occurring only in endemic areas (Kano et al., 2012; Griffin et al., 2015; Stanisic et al., 2015; França et al., 2016; França et al., 2017; He et al., 2019; Kale et al., 2019). Longley et al. suggest the use of antibodies against PvEBP-2 as a possible serological marker to detect recent infection by *P. vivax* (Longley et al., 2020). This data reinforces the notion that NP is constantly exposed to malaria transmission in the Atlantic Forest in Brazil.

*Plasmodium simium*-infected NP had a high seroprevalence of IgM against PvCSP, PvAMA-1, and PvDBPII, as well as high levels of IgM antibodies against PvEBP-2 and PvDBPII. A positive correlation was previously reported for antibody responses (either IgM or IgG) against PvAMA-1 and malaria positivity (Rodrigues et al., 2005; Kale et al., 2019). The absence of a correlation between anti-PvDBPII and PvEBP-2 IgM in acute malaria infections was observed in humans from the Amazon and areas outside the Amazon areas (Medeiros et al., 2020; Lima et al., 2022). Significant differences were not observed in either the seroprevalence or the levels of IgM between the different sexes of NP. In general, there are no great differences in the behavior of male and female brown howler monkeys in the wild, such as mobility, which would imply that both have the same exposure to malaria parasites (Crockett and Eisenberg, 1987; Neville et al., 1988; Rudran and Fernandez-Duque, 2003).

Overall, here, we observed a high frequency of NP from each taxonomic family responding to CSP peptides, but most of them showed only low levels of antibodies. Conversely, almost all animals showed IgM against the studied erythrocytic stage antigens, many with high levels, regardless of their taxonomic family. Previous studies have shown the presence of IgG antibodies against CSP and different erythrocytic stage antigens of *P. vivax, P.malariae/P. brasilianum*, and *P. falciparum* mainly in *Alouatta guariba clamitans* of the Atelidae family, from the Atlantic Forest areas of the states of São Paulo, Rio de Janeiro, and Santa Catarina (Duarte et al., 2006; Yamasaki et al., 2011; Costa et al., 2014; Monteiro et al., 2020; de Assis et al., 2021). Our group previously reported IgG antibodies against CSP peptides and erythrocytic stage antigens among NP of the Cebidae and Callitrichidae families from the state of Rio de Janeiro state; however, similar results could not be shown for these NP families from the Atlantic Forest in the states of São Paulo (Duarte et al., 2006; Yamasaki et al., 2011; Monteiro et al., 2020; de Assis et al., 2021). Nevertheless, the IgG profile was the opposite of the IgM profile for these families, with high response against CSP peptides for all families, but high IgG levels for erythrocytic stage antigens mainly for the Atelidae family (de Assis et al., 2021). Overall, the IgM and IgG results suggested that all NP families have similar frequencies of both IgM and IgG, suggesting that all of them have been constantly exposed to infected mosquito bites. However, the intensity of responses (as measured by the reactivity index) shows differences among NP families, with the Atelidae family having the highest levels of anti-CSP IgG, and high IgM and IgG against erythrocytic stage antigens, suggesting that well-established *Plasmodium* infections frequently and mostly occur in this NP family. However, we are aware of the limitations of this conclusion due to the small sample size per family.

Interestingly, generalized linear models allowed us to demonstrate that the major variables (age, sex, free-living NP, and PCR positivity) responsible for the IgM response identified here were the presence of PCR-detectable *Plasmodium* infection and being free-living animals. NP with active infection showed more chances of having IgM against CSP peptides compared to uninfected animals, mainly IgM against PvCSP. No difference was observed in the seroprevalence against CSP in infected and uninfected humans from malaria endemic area (Pereira et al., 2018). Free-living NP also showed a higher chance of having IgM against *Plasmodium* CSP, especially PfCSP. Moreover, free-living animals showed more chances of an IgM response against erythrocytic stage antigens, mainly PvAMA-1, compared to captive animals. PvAMA-1 was highly immunogenic in humans exposed to multiple malaria infections in Brazil and India (Rodrigues et al., 2005; Kale et al., 2019). Overall, these findings suggest that IgM response against PvCSP and PvAMA-1 may be predictors of the potential reservoirs of malaria in the Atlantic Forest. In comparison, free-living NP was more likely to have IgG against both pre-erythrocytic, mainly PvCSP, and erythrocytic stage proteins, mainly PvDBPII (**Supplementary Figure 7**).

We observed a higher frequency of IgM in NP with PCR-detectable malaria infection compared to uninfected individuals, in addition to an association between IgM response to PvCSP and PVAMA-1 and active infection. This raises the question of whether the IgM antibodies in these NP were recently acquired or not. Recent studies demonstrate that IgM-expressing memory B cells are expanded in humans living in malaria-endemic areas (Krishnamurty et al., 2016). Additionally, parasite-specific IgM was recently shown to persist for a long time in patients exposed to *P. vivax* transmission (Patgaonkar et al., 2018; Medeiros et al., 2020). Boyle et al. detected *P. falciparum*-specific IgM antibodies more than 6 months after *Plasmodium* infection in Australians returning from malaria-endemic areas (Boyle et al., 2019). All these findings of long-term IgM responses emphasize the need to better understand the meaning of specific IgM antibodies in malaria infection. In non-human primates, both specific and total IgM antibodies against infected red blood cells (iRBC) were shown to increase and remain elevated during and after the peak of primary infection in the *Plasmodium cynomolgi*-rhesus macaque model (Joyner et al., 2019). Moreover, an inverse association between specific IgM antibodies and parasitemia during relapse has suggested that these antibodies may play a role in neutralizing blood-stage parasites. One limitation of the current study is our focus only on seroepidemiology based on ELISA results, not including functional assays, such as merozoite invasion and cellular lysis. Nonetheless, a follow-up study on IgM in non-human primates naturally-exposed to *Plasmodium* sp. is still required to confirm these findings and evaluate the dynamics of acquisition and persistence of this antibody class.

Our findings suggest that NP from distinct Atlantic Forest areas have had natural exposure to sporozoites and often developed erythrocytic stage infections with different species of *Plasmodium*. For the first time, the humoral IgM immune response was evaluated in naturally infected NP. IgM had significant associations with free-living animals and established malaria infection, mainly for antibodies against PvCSP and PvAMA-1. Thus, the presence of IgM antibodies against these antigens could be used as predictors of NP potentially acting as reservoirs of malaria in the Brazilian Atlantic Forest.

## Data availability statement

The original contributions presented in the study are included in the article/**Supplementary material**. Further inquiries can be directed to the corresponding author.

## Ethics statement

The animal study was reviewed and approved by Fiocruz Research Ethical Committee (CEUA license L037/2016) and by the Brazilian Ministry of Environment (SISBIO numbers 43375-4, 43375-6, 54707-137362-2, 52472-1, 28953-1).

## Author contributions

CB, LC, and GA designed and conceived the study. GA, DA, AP-C, JJ, GG, AN, AP, SM, HT, VP, and ZH were responsible for sample collection. LT, HC, FK, IS, FN, JA, JF, and LP-R were responsible for the production of *Plasmodium* proteins and peptides. GA, DA, and LS performed the experiments. ES performed the statistic model. GA, DA, JS-A, TS, FK, LC, LP-R, CD-R, and CB analyzed and interpreted data. GA, DA, and CB wrote the first draft. All authors contributed to the article and approved the submitted version.
