# Supplementary material for: IgM antibody responses against Plasmodium antigens in neotropical primates in the Brazilian Atlantic Forest
Source: Front Cell Infect Microbiol. 2023 Sep 27;13:1169552. doi: 10.3389/fcimb.2023.1169552 (PMC10565664; doi:10.3389/fcimb.2023.1169552)
Supplement: Supplementary file 1 [file DataSheet_1.docx]

Supplementary Material

IgM antibodies response against *Plasmodium* antigens in neotropical primates in the Brazilian Atlantic Forest

Gabriela Maíra Pereira de Assis^1^, Denise Anete Madureira de Alvarenga^1^, Luisa Braga e Souza^1^, Juan Camilo Sánchez-Arcila^2^, Eduardo Fernandes e Silva^3^, Anielle de Pina-Costa^,4,5,6,7^, Gustavo Henrique Pereira Gonçalves^8,9^, Júlio César de Souza Junior^8^, Ana Julia Dutra Nunes^8,10,9^, Alcides Pissinatti^11,12^, Silvia Bahadian Moreira^11^, Leticia de Menezes Torres^1^, Helena Lott Costa^1^, Herlandes da Penha Tinoco^13^, Valéria do Socorro Pereira^13^, Irene da Silva Soares^14^, Taís Nóbrega Sousa^1^, Francis Babila Ntumngia^15^, John H. Adams^15^, Flora Satiko Kano^1^, Zelinda Maria Braga Hirano^7,8^, Lilian Rose Pratt Riccio^6^, Cláudio Tadeu Daniel-Ribeiro^5,6^, Joseli Oliveira Ferreira^16^, Luzia Helena Carvalho^1^, Cristiana Ferreira Alves de Brito^1*^

*** Correspondence:** Cristiana Ferreira Alves de Brito. cristiana.brito@fiocruz.br

**
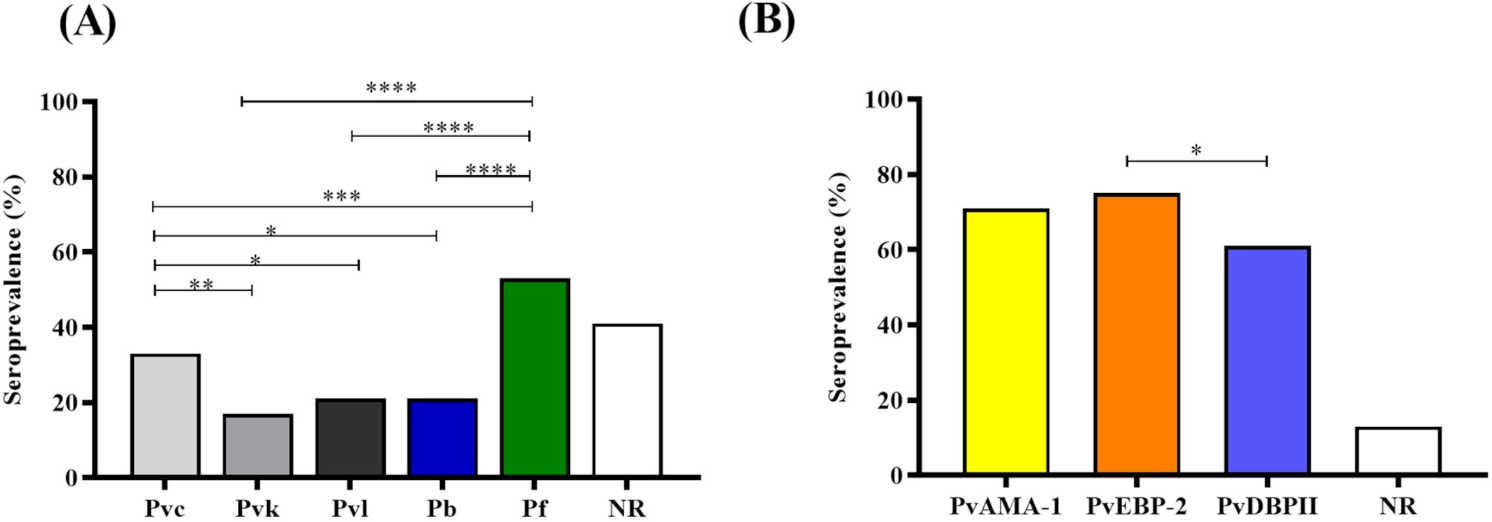
**

**Supplementary Figure 1 – Seroprevalence of IgM antibodies against *Plasmodium* antigens of neotropical primates from all three studied areas.** Seroprevalence of IgM antibodies against **(A)** circumsporozoite protein (CSP) peptides and **(B)** *Plasmodium vivax* erythrocytic stage antigens. Pvc: *P.vivax* CSP classic VK210 variant (light grey); Pvk: *P. vivax* CSP VK247 variant (grey); Pvl: CSP *P.vivax-*like variant (black); Pb/Pm: *P. brasilianum/P. malariae* CSP (blue); Pf: *P. falciparum* CSP (green); NR: Non-responder (white); PvAMA-1: *P. vivax* Apical Membrane Antigen 1 (yellow); PvEBP-2: *P. vivax* Erythrocyte Binding Protein 2 (Orange); PvDBPII*:* domain II of the *P. vivax* Duffy Binding Protein (blue). (**P* < 0.05; ***P* < 0.01; ****P* < 0.001; *****P* < 0.0001).


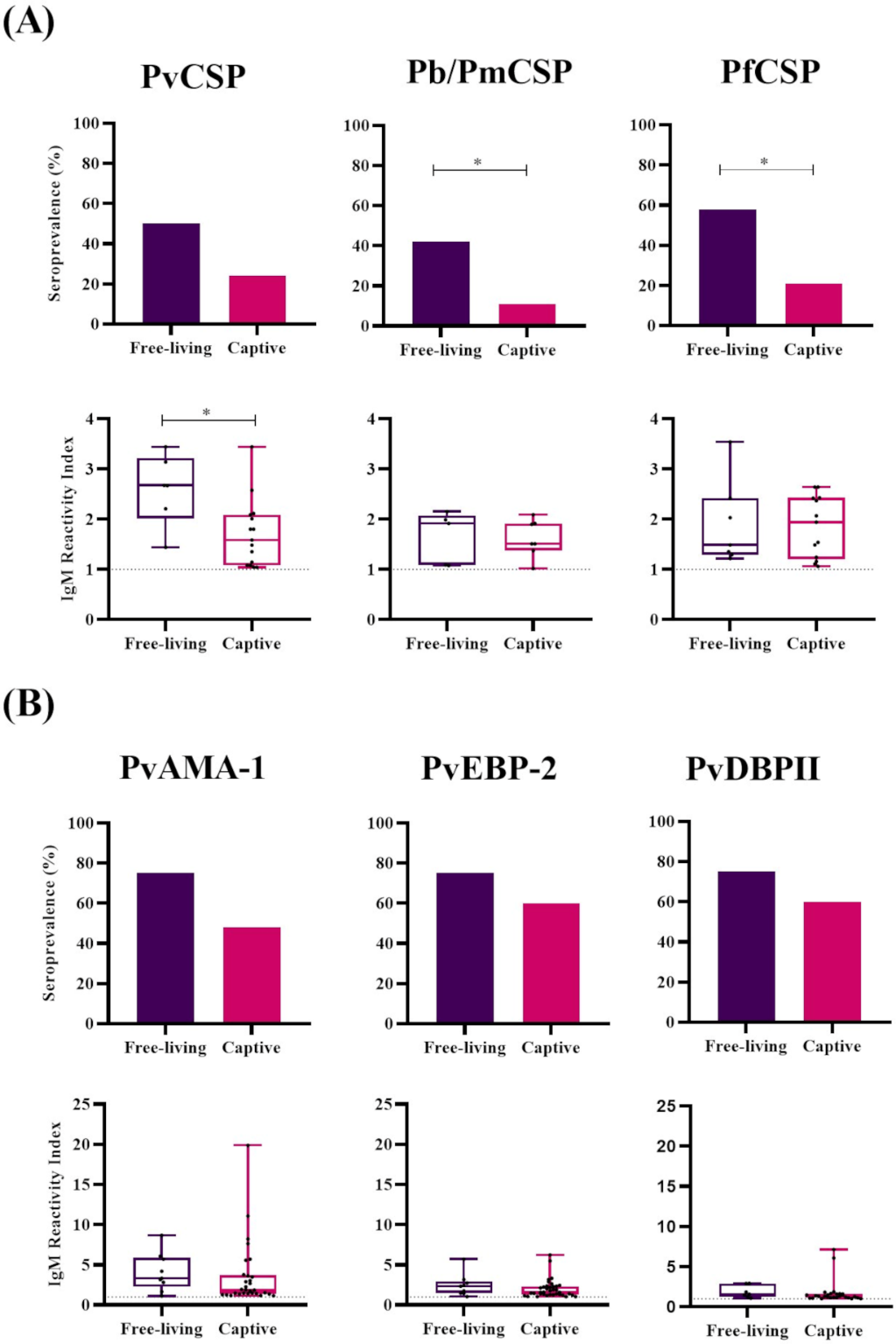


**Supplementary Figure 2 - Seroprevalence and levels of antibodies against *Plasmodium* antigens in Free-living and Captive animals from Indaial/SC.** Seroprevalence and reactivity index (RI) of IgM antibodies against **(A)** circumsporozoite protein (CSP) peptides and **(B)** *P. vivax* erythrocytic stage antigens. RI > 1 was considered positive (dotted line). The filled bars indicate the percentage of NP with RI > 1. Data expressed as individual RI values (dots) and median with the interquartile range (boxes). PvCSP: *P. vivax* CSP variants (VK210, VK247, and/or *P. vivax-like*); Pb/PmCSP: CSP repeat of *P. brasilianum/P. malariae*; PfCSP: CSP repeat of *P. falciparum*; PvAMA-1: *P. vivax* Apical Membrane Antigen 1; PvEBP-2: *P. vivax* Erythrocyte Binding Protein 2; PvDBPII: domain II of the *P. vivax* Duffy Binding Protein region. Free-living: purple; Captive: pink. Differences statistically significant were indicated by asterisk (**P* < 0.05).


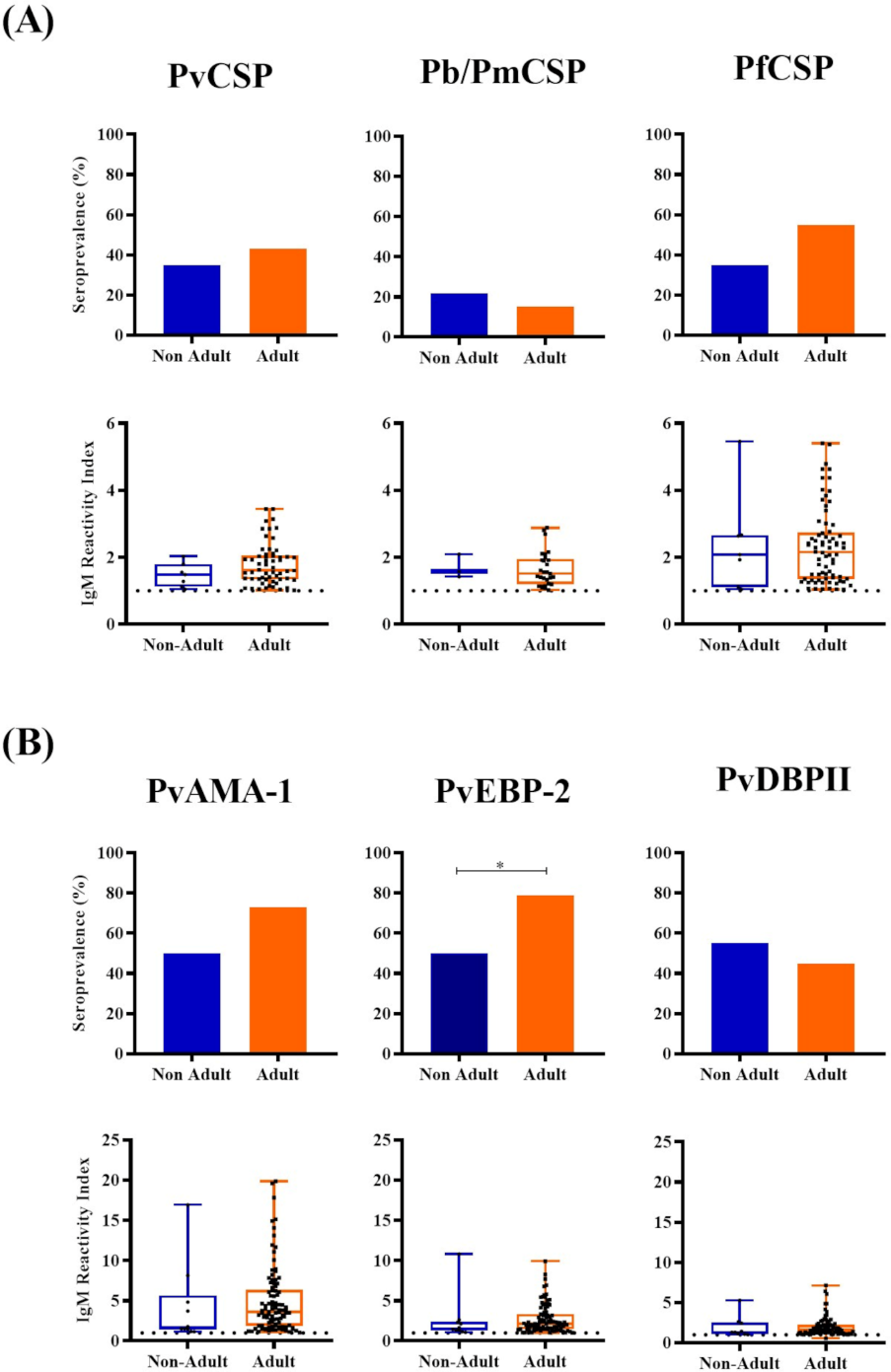


**Supplementary Figure 3 - Seroprevalence and levels of IgM antibodies against *Plasmodium* antigens according to the age of Neotropical primates from Indaial/SC and Joinville/SC. (A)** Seroprevalence and reactivity index (RI) of IgM antibodies against circumsporozoite protein (CSP) peptides. **(B)** Seropositivity and RI of IgM antibodies against *Plasmodium vivax* erythrocytic stage antigens**.** RI > 1 was considered positive (dotted line). The filled bars indicate the percentage of NP with RI > 1. Data expressed as individual RI values (dots) and median with the interquartile range (boxes). Age estimated according to **Carpenter, 1965**, and categorized in non-Adult (blue) and Adult (orange). PvCSP: *P. vivax* CSP variants (VK210, VK247, and/or *P. vivax-like*); Pb/PmCSP: CSP repeat of *P. brasilianum/P. malariae*; PfCSP: CSP repeat of *P. falciparum*; PvAMA-1: *P. vivax* Apical Membrane Antigen 1; PvEBP-2: *P. vivax* Erythrocyte Binding Protein 2; PvDBPII: domain II of the *P. vivax* Duffy Binding Protein. Differences statistically significant were indicated by asterisk (**P* < 0.05).


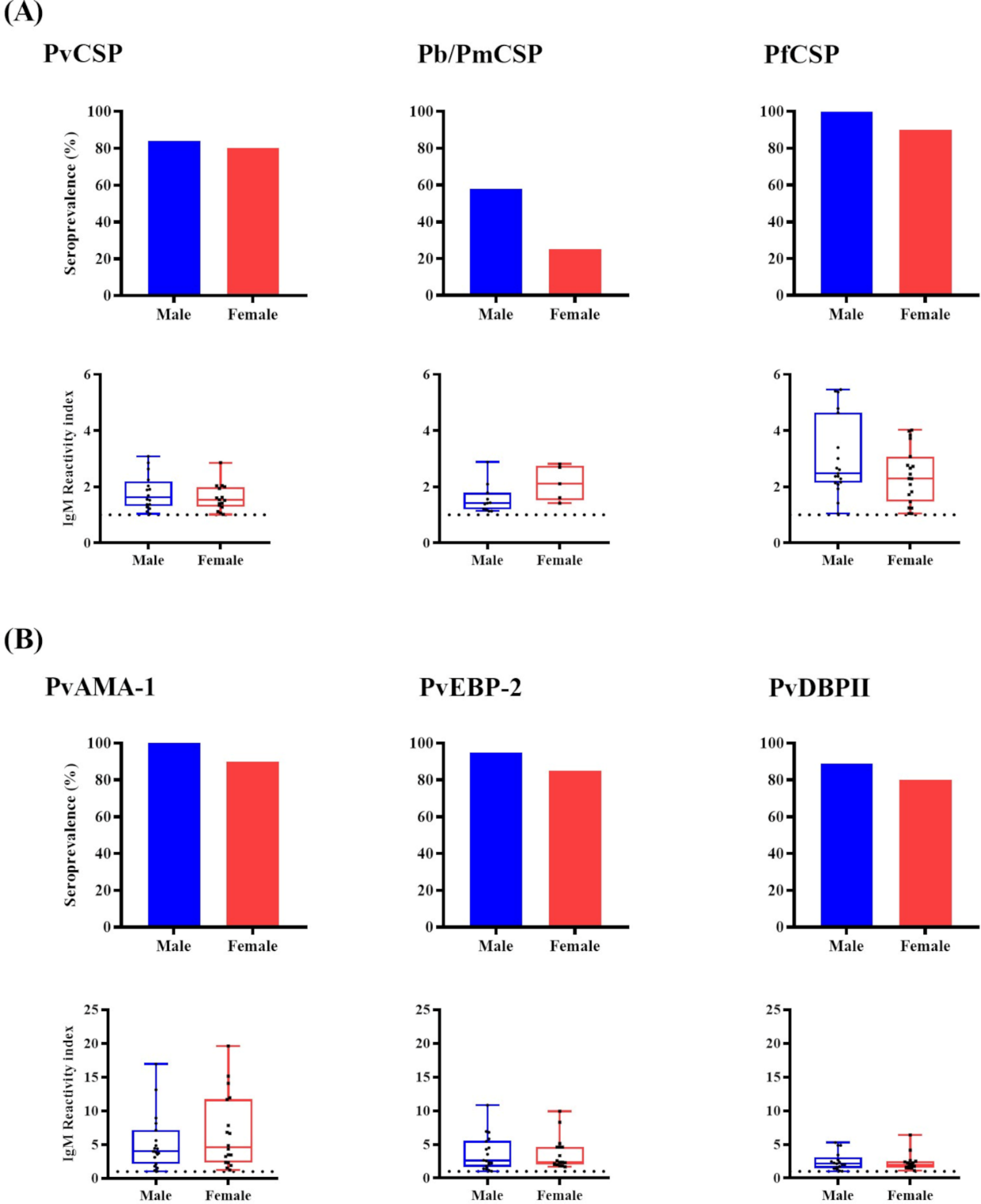


**Supplementary Figure 4** - **Association between IgM antibody responses and sex in free-living animals from Joinville/SC. (A)** Seroprevalence and reactivity index (RI) of IgM antibodies against circumsporozoite protein (CSP) repeats. **(B)** Seroprevalence and RI of IgM antibodies against *Plasmodium vivax* erythrocytic stage antigens. RI>1 was considered positive (dotted line). The filled bars indicate the percentage of NP with RI>1. Data expressed as individual RI values (dots) and median with the interquartile range (boxes). PvCSP: *P. vivax* CSP variants (VK210, VK247, and/or *P. vivax*-like); Pb/PmCSP: CSP repeat of *P. brasilianum/P. malariae*; PfCSP: CSP repeat of *P. falciparum*; PvAMA-1: *P. vivax* Apical Membrane Antigen 1; PvEBP-2: *P. vivax* Erythrocyte Binding Protein 2; PvDBPII: domain II of the *P. vivax* Duffy Binding Protein. Male: blue; Female: red. No comparison showed a statistically significant difference.


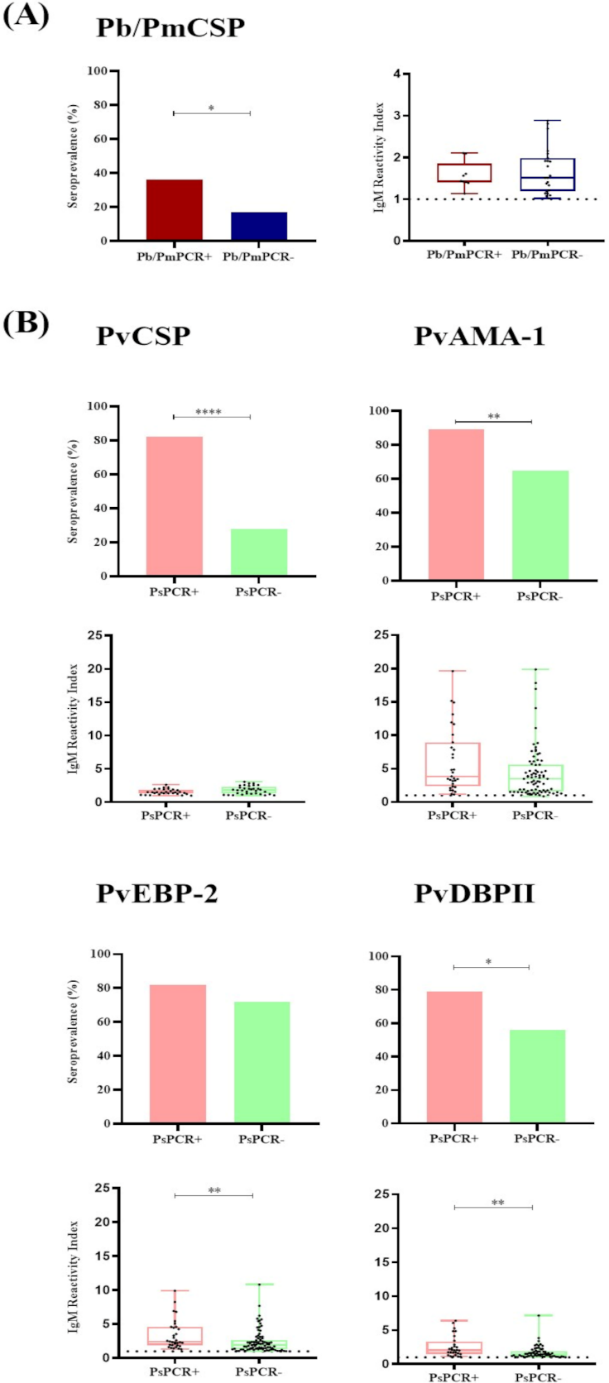


**Supplementary Figure 5** - **Association between IgM responses according to active *Plasmodium* infection of animals from all three studied areas. (A)** Seroprevalence and reactivity index (RI) of IgM antibodies against *P. brasilianum/P. malariae* circumsporozoite protein (CSP) peptide in monkeys with PCR positive for *P. brasilianum/P. malariae* (Pb/PmPCR+, red) or negative (Pb/PmPCR-, blue)*.* **(B)** Seroprevalence and RI of IgM antibodies against *P. vivax* CSP and erythrocytic stage antigens in monkeys with PCR positive for *P. simium* (PsPCR+, salmon) or negative (PsPCR-, light green)*.* RI > 1 was considered positive (dotted line). The filled bars indicate the percentage of NP with RI > 1. Data expressed as individual RI values (dots) and median with the interquartile range (boxes). Pb/PmCSP: CSP repeat of *P. brasilianum/P. malariae*; PvCSP: *P. vivax* CSP repeats (VK210, VK247, and/or *P. vivax*-like); PvAMA-1: *P. vivax* Apical Membrane Antigen 1; PvEBP-2: *P. vivax* Erythrocyte Binding Protein 2; PvDBPII: domain II of the *P. vivax* Duffy Binding Protein. Differences statistically significant were indicated by asterisk (**P* < 0.05; ***P* < 0.01; *****P* < 0.0001).


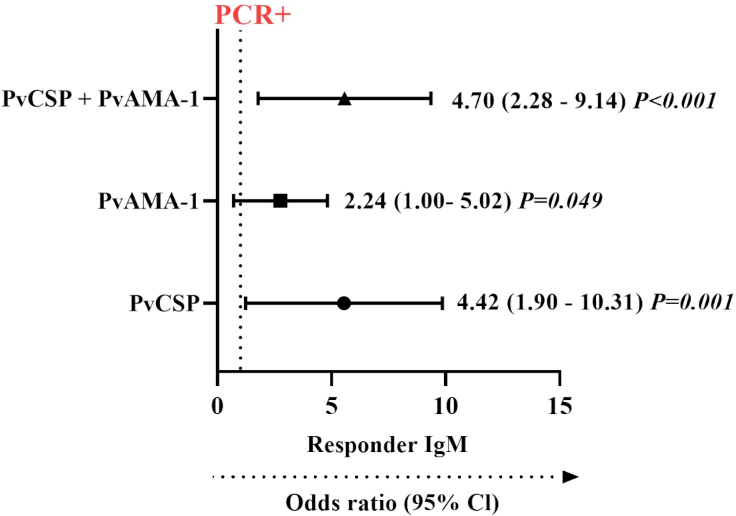


**Supplementary Figure 6 - Odds ratio for IgM antibody responses of Neotropical primates against circumsporozoite protein (CSP) and/or AMA-1 of *Plasmodium vivax* according to PCR positivity.** PvCSP: *P. vivax* CSP variants (VK210, VK247, and/or *P. vivax-like);* PvAMA-1: *P. vivax* Apical Membrane Antigen 1. The data shown are only the significant values for the odds ratios (logistic Regression, *P*<0.05).


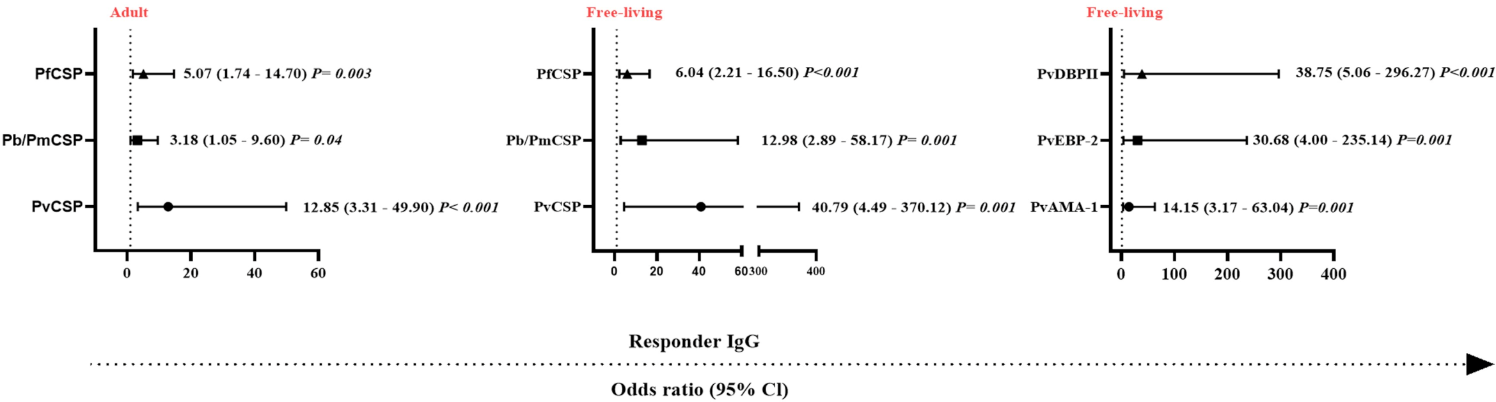


**Supplementary Figure 7 – Odds ratios for IgG antibody responses of Neotropical primates against different *Plasmodium* antigens according to different sampling parameters*.*** Comparison of the odds ratio for seroprevalence of IgG against various circumsporozoite protein (CSP) peptides from different Plasmodium species and *P. vivax* erythrocytic stage antigens according to NP age (adult or non-adult) and whether sampled individuals were either free-living or captive. PvCSP: *P. vivax* CSP variants (VK210, VK247, and/or *P. vivax-like*); Pb/PmCSP: CSP repeat of *P. brasilianum/P. malariae*; PfCSP: CSP repeat of *P. falciparum*; PvAMA-1: *P. vivax* Apical Membrane Antigen 1; PvEBP-2: *P. vivax* Erythrocyte Binding Protein 2; PvDBPII: domain II of the *P. vivax* Duffy Binding Protein. The data shown are only the significant values for the odds ratios (logistic Regression, *P*<0.05). The free-living versus captive comparison was performed only for the group of NP from Indaial/SC.
